# Characteristic Extraction and Assessment Methods for Transformers DC Bias Caused by Metro Stray Currents

**DOI:** 10.3390/e26070595

**Published:** 2024-07-11

**Authors:** Aimin Wang, Sheng Lin, Guoxing Wu, Xiaopeng Li, Tao Wang

**Affiliations:** 1 School of Electrical Engineering and Electronic Information, Xihua University, Chengdu 610039, China; 1220210049@mail.xhu.edu.cn (A.W.); twang@mail.xhu.edu.cn (T.W.); 2 School of Electrical Engineering, Southwest Jiaotong University, Chengdu 611756, China; 3 Shenzhen Power Supply Bureau Co., Ltd., China Southern Power Grid, Shenzhen 518000, China; wuguoxing@sz.csg.cn; 4 State Grid Sichuan Electric Power Research Institute, Chengdu 610044, China; lixp6053@sc.sgcc.com.cn

**Keywords:** metrostray current, transformers DC bias, characteristic extraction, risk assessment, neutral DC, vibration

## Abstract

Metro stray currents flowing into transformer-neutral points cause the high neutral DC and a transformer to operate in the DC bias state.Because neutral DC caused by stray current varies with time, the neutral DC value cannot be used as the only characteristic indicator to evaluate the DC bias risk level. Thus, unified characteristic extraction and assessment methods are proposed to evaluate the DC bias risk of a transformer caused by stray current, considering the signals of transformer-neutral DC and vibration. In the characteristic extraction method, the primary characteristics are obtained by comparing the magnitude and frequency distributions of transformer-neutral DC and vibration with and without metro stray current invasion. By analyzing the correlation coefficients, the final characteristics are obtained by clustering the primary characteristics with high correlation. Then, the magnitude and frequency characteristics are extracted and used as indicators to evaluate the DC bias risk. Moreover, to avoid the influence of manual experience on indicator weights, the entropy weight method (EWM) is used to establish the assessment model. Finally, the proposed methods are applied based on the neutral DC and vibration test data of a certain transformer. The results show that the characteristic indicators can be extracted, and the transformer DC bias risk can be evaluated by using the proposed methods.

## 1. Introduction

Since the metro rail cannot completely insolate to the soil, DC in the rail leaks from it and forms the metro stray current [1]. Metro stray currents flowing into substations near the metro line cause the transformer-neutral DC to increase and facilitate the transformer to operate in the DC bias state [2]. DC bias can lead to an increase in transformer vibration, threatening the safety of the mechanical structure of a transformer [3]. Because high neutral DC is the reason for DC bias, the magnitude of neutral DC is always used as the critical indicator to identify and evaluate the DC bias risk in the field [4]. However, the neutral DC caused by metro stray current fluctuates with time positively and negatively, which is affected by the alternate change between metro train traction and braking [1]. By continuing to use the neutral DC magnitude as the only indicator to evaluate the transformer DC bias risk, the results could vary dramatically with time and be inaccurate because the neutral DC is not always kept at the magnitude level. Therefore, besides neutral DC, the characteristics of vibration may help to assess the risk level of transformer DC bias caused by metro stray currents.

Based on field experience, once the neutral DC value exceeds the threshold, the transformer is considered to operate in the DC bias state [4]. However, the neutral DC caused by stray currents varies with time positively and negatively [5]. Thus, the DC bias may also exist even if the neutral DC is less than the threshold value at present. Moreover, high neutral DC may not be the first aspect tested or concerned; the transformer vibration caused by high neutral DC is commonly the first signal to be noticed by substation staff [4]. Moreover, the measurement method of transformer vibration is simple and easy to implement because the vibration sensor does not need to intrude into the transformer or be electromagnetically coupled to the transformer circuit [6]. Thus, considering the effect of neutral DC on transformer vibration, the vibration characteristics have been analyzed and used to identify the transformer DC bias [7]. By analyzing the frequency distribution of transformer vibration based on the Fourier transform method, the odd-even frequency ratio, frequency complexity, and distortion rate are proposed as frequency indicators to identify transformer DC bias [8,9,10,11]. Based on time-frequency transform methods, such as wavelet transform and empirical modal decomposition, the time-frequency distribution characteristics of transformer vibration are obtained and analyzed under the DC bias state [12]. The time-frequency indicators, such as wavelet entropy, strange entropy, and time-frequency map, are proposed to identify transformer DC bias [4,13,14]. Then, based on the proposed indicators, transformer DC bias is identified by using machine learning or artificial intelligence algorithms [15,16,17].

Since transformer vibration relates to the neutral DC level based on electromagnetic theory, the neutral DC and vibration can be used to evaluate the DC bias risk level. At present, the evaluation methods for DC bias caused by the ground electrode currents of high-voltage DC systems (HVDC) have been proposed [18,19]. According to DC bias reasoning, the higher the neutral DC magnitude, the higher the risk of DC bias [7]. However, because the neutral DC of a transformer caused by the HVDC ground electrode current is commonly constant, the transformer vibration is used to evaluate the DC bias risk level [17]. The fuzzy-generating functions were used to quantify the vibration. Then, the DC bias risk is evaluated based on expert experience, the fuzzy method, and the analytic hierarchy process method [13,18,19]. To this extent, when using the vibration signal to assess the DC bias risk, the natural DC is always ignored. However, the neutral DC caused by stray currents is not constant [1]. The fluctuation of stray currents may affect the transformer vibration characteristics. Thus, the neutral DC and vibration should both be used to assess the DC bias risk caused by stray currents.

In order to evaluate the transformer DC bias risk caused by stray current, a unified characteristic extraction and assessment method is proposed based on transformer-neutral DC and vibration. In the characteristic extraction method, time-magnitude and time-frequency distributions of neutral DC and vibration for transformers with and without metro stray current interferences are compared. Then, by considering the synchronization of transformer-neutral DC and vibration, the magnitude and frequency characteristic indicators are extracted and combined based on the correlation coefficient and synthetic hierarchical clustering algorithm. In the assessment method, to avoid the influence of manual experience on indicator weights, the entropy weight method (EWM) is used to establish the assessment model. Based on the test data of transformer-neutral DC and vibration, the proposed characteristic extraction method is applied, and the DC bias risk is evaluated using the proposed assessment model.

## 2. Characteristic Extraction Method

### 2.1. Characteristic Mechanism

Based on electromagnetic theory, the vibration acceleration of a transformer relates to the neutral DC level as follows:

(1) $$a=\frac{-2\varepsilon_{s}N^{2}}{H_{c}^{2}l^{2}L^{2}}\left[I_{0}U_{m}\cos\left(k\omega t+\varphi \right)+U_{m}^{2}\cos\left(2k\omega t+2\varphi \right)\right]$$

where *a* is the vibration acceleration; *N* is the number of winding turns; *l* is the number of integral paths; *L* is the silicon steel sheet dimensions; $\varepsilon_{s}$ is the magnetostriction ratio of silicon steel sheet; $I_{0}$ is the DC flowing into the transformer; $U_{m}$ is the AC voltage of the transformer.

According to the function, vibration acceleration and odd-frequency components both increase with DC magnitude increases when DC flows into transformer winding. Thus, the magnitude and frequency characteristics of transformer-neutral DC and vibration can be used as indicators to assess the DC bias risk level.

### 2.2. Method Principle

**Figure 1:**
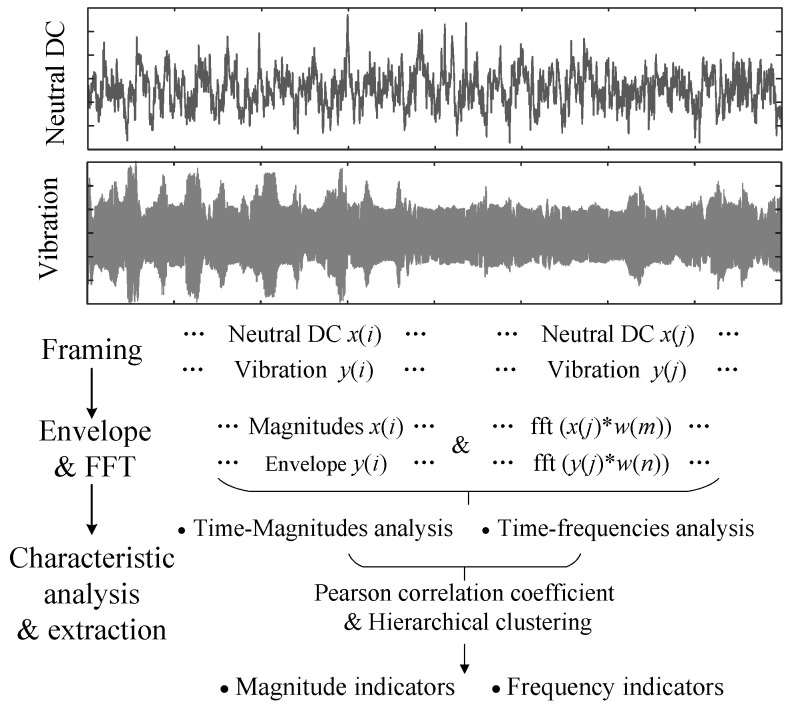
Method principle of characteristic analysis and extraction.

In order to obtain the magnitude and frequency characteristics of transformer-neutral DC and vibration, signal analysis and extraction steps are necessary. The method principle proposed by this research is shown in Figure 1. There are two steps before characteristic analysis and extraction, including data preprocessing and conversion. In the data preprocessing process, the data discretization is realized by using a frame operation. In the data-conversion process, the time-magnitude and time-frequency distributions are obtained by envelope calculations and fast Fourier transform. Then, by analyzing the magnitude and frequency characteristics, the characteristic indicators are proposed using the Pearson correlation coefficient and hierarchical clustering methods.

### 2.3. Data Preprocessing and Conversion

#### 2.3.1. Data Preprocessing

In order to analyze the magnitude and frequency characteristics, the first step is to preprocess the test data, including framing and windowing. The first step is to discrete the test data considering different time scales and analysis objectives. In order to obtain the magnitude information, the test date is discrete with a time scale of 1 s. When analyzing the frequency characteristics, the time headway is selected as the framing time considering train operation.

After the framing step with 1 s, the neutral DC is expressed as x(i), and the vibration is y(i), where 1≤i≤N and *N* is the round down of the test time length. After the framing step with the train time headway, the neutral DC is x(j), and the vibration is y(j), where 1≤j≤f(N/T), f(N/T) is the round down of N/T, and *T* is the train time headway.

#### 2.3.2. Data Conversion

The time-magnitude distributions of neutral DC and vibration are obtained based on the data x(i) and y(i). The average value x(i) is the neutral DC magnitude in the time *i*. The upper and lower envelopes of y(i) are the vibration magnitudes. Then, the time-magnitude distribution is obtained by arranging the neutral DC and vibration magnitudes with time.

The time-frequency distribution is obtained by pulsing the Hamming window function and fast Fourier transform based on the data x(j) and y(j). The hamming windows ω(m) and ω(n) are selected as the window function, where *m* and *n* are the sampling numbers of neutral DC and vibration. Because the frequency range of neutral DC is mostly from 0 to 0.1 Hz, the frequency components from 0 to 0.1 Hz are extracted as the frequency characteristics. Since the main components of the vibration signal shown in function (1) is a multiple of 50 Hz, the frequency multiplications are extracted as the frequency characteristics. Then, the time-frequency distribution of each frequency multiplication is obtained by arranging the frequency multiplication amplitude with time.

### 2.4. Characteristic Analysis and Extraction

#### 2.4.1. Characteristic Analysis

**Figure 2:**
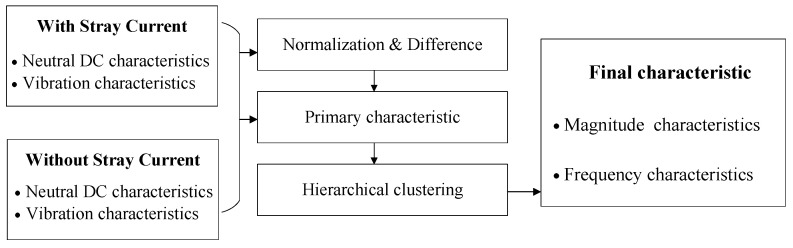
Method flow chart of characteristic analysis.

The characteristic analysis steps are shown in Figure 2. According to the magnitude and frequency distributions of neutral DC and vibration for a transformer with stray current flowing and no stray current flowing, the normalization and difference calculations are performed. The characteristics corresponding to large differences are extracted and selected as the primary characteristics. The normalization method is as follows:

(2) $$\tilde{x_{i}}=\frac{x_{i}}{{∥X∥}_{2}},x_{i}\in X$$

where $x_{i}$ is the magnitudes or the frequencies of neutral DC or vibration after data conversion; *X* is the matrix of the magnitude or frequency data; ${∥\cdot ∥}_{2}$ is used to calculate the 2-norm of the matrix.

To this extent, the number of primary characteristics is large, and using these primary characteristics may make it difficult to quantify and evaluate the DC bias risk. Moreover, among these primary characteristics, some characteristics may change synchronously. Thus, the number of characteristics can be reduced by combining these features.

The Pearson correlation coefficient is used to combine the primary characteristics. First, the Pearson correlation coefficients among the primary characteristics are calculated as follows:

(3) $$r=\frac{\sum_{i}^{N}\left(x_{i}-\bar{X}\right)\left(y_{i}-\bar{Y}\right)}{\sqrt{\sum_{i}^{N}\left(x_{i}-\bar{X}\right)}\sqrt{\sum_{i}^{N}\left(y_{i}-\bar{Y}\right)}}$$

where $x_{i}$, $y_{i}$, $\bar{X}$, and $\bar{Y}$ are the primary characteristics and average values.

Then, the hierarchical clustering method is used to combine the characteristics with a high Pearson correlation coefficient. The Pearson correlation coefficients are considered as the distance between two characteristics. In the combining process of similar characteristics, the number of combined characteristics is determined by the correlation coefficient threshold. The combined characteristics are the final magnitude and frequency characteristics to be used to extract the evaluation indicators of DC bias risk.

#### 2.4.2. Indicator Extraction

Based on the final characteristics, the amplitude of magnitude characteristics are extracted as magnitude indicators. The energies of the frequency characteristics are extracted as frequency indicators.

- Magnitude indicator *M*

Based on the neutral DC magnitude and vibration envelopes, the magnitude indicator *M* is proposed to represent the amplitude characteristic. The magnitude indicator *M* is calculated as follows:

(4) $$M=\sqrt{\frac{1}{N}\sum_{i=1}^{N}x_{i}^{2}}$$

where $x_{i}$ is the neutral DC magnitude or vibration envelope. *N* is the number of sampling data.

- Frequency indicator $F_{k}$

Based on the frequency characteristics of neutral DC and vibration, the frequency indicator $M_{1}$ is proposed to represent the energy proportion of the frequency component. The frequency indicator $F_{k}$ is calculated as follows:

(5) $$F_{k}=\frac{f_{k}^{2}\;}{\sum_{i=1}^{N_{1}}f_{i}^{2}}$$

where $f_{k}$ is the frequency component from the final characteristic frequencies; $f_{i}$ is the frequency component from the frequency multiplications of 50 Hz after data conversion; $N_{1}$ is the number of frequency multiplications.

## 3. Assessment Method of DC Bias Risk

### 3.1. Assessment Principle

**Figure 3:**
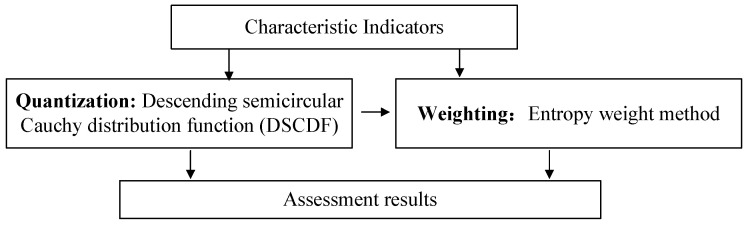
Method principle of DC bias risk assessment.

The assessment method principle of transformer DC bias risk is shown in Figure 3. Three steps are needed in the method, including indicator quantization, indicator weighting, and assessment result calculation. In the quantization step, the smaller the indicator value, the smaller the transformer DC bias risk. Thus, the descending semicircular Cauchy distribution function (DSCDF) is used to quantize the indicator. Then, considering the expert experience and characterization fluctuations, EWM is used to calculate the indicator weighting. The higher the calculation value, the larger the transformer DC bias risk.

### 3.2. Indicator Quantization

The DSCDF is used to quantize the indicator as follows:

(6) $$f\left(x\right)=\left\{\begin{array}{cc} 1 & x\leq x_{th}\\ \frac{1}{1+{\left(x-x_{th}\right)}^{2}} & x>x_{th} \end{array}\right.$$

where f(x) is the evaluation result of the indicator *x*; *x* is the indicator value; $x_{th}$ is the threshold value of the indicator, which can be set according to engineering experience or standards; By using the DSCDF, the evaluation results of the indicator range are set as (0, 1].

### 3.3. Indicator Weighting

The indicators are from different signals and domains. When using EWM, the first process is to normalize the indicator. Then, the weight of each indicator is calculated as follows:

(7) $$p_{ij}=\frac{x_{j}\left(i\right)}{\sum_{i=1}^{N_{0}}x_{j}\left(i\right)}$$

(8) $$H_{j}=-\frac{1}{\ln N_{0}}\left(\sum_{i=1}^{N_{0}}p_{ij}\ln p_{ij}\right)$$

(9) $$\omega =\frac{1-H_{j}}{n-\sum_{j=1}^{Q}H_{j}}$$

where $p_{ij}$ is the proportionate value of the normalization indicator $x_{j}\left(i\right)$; *j* is the indicator type; $N_{0}$ is the sample number of indicator $x_{j}$; $H_{j}$ is the entropy value of indicator type $x_{j}$; ω is the weighting values matrix; *Q* is the indicator types.

### 3.4. Comprehensive Assessment

The DC bias risk assessment includes two steps. The first step is to calculate the indicator scores using DSCDF. The second step is to calculate the DC bias risk.

According to the indicator score results calculated by using DSCDF, the assessment scores of DC bias risk *O* are calculated as follows:

(10) $$O=\sum_{i=1}^{n}\omega_{i}\left(1-f\left(x_{i}\right)\right)$$

where $f(x_{i})$ is the score of indicator $x_{i}$, calculated by using DSCDF, $0\leq f(x_{i})<1$. The larger the assessment result *O*, the higher the DC bias risk. Based on engineering experience, the transformer DC bias risk levels are classified based on Table 1.

By using DSCDF, the neutral DC and vibration indicators are normalized, which gives the characteristics the same metric scale. Compared with the analytic hierarchy process and other expert scoring methods, the proposed evaluation method is simple, easy to use, and relatively comprehensive. Moreover, EWM is not affected by subjective factors, considering the relationship between the indicators, and is suitable for data analysis.

## 4. Method Application

For a certain 220 kV substation, the transformer-neutral DC and vibration are tested with and without the metro stray current effect. The neutral DC is tested by using the DC hall sensor with a sampling frequency of 2 Hz. Considering the frequency range of transformer vibration is mostly from 0 to 600 Hz, the vibration is measured by using a vibration acceleration sensor with a sampling frequency of 1200 Hz.

### 4.1. Characteristic Extraction of the Test Data

**Figure 4:**
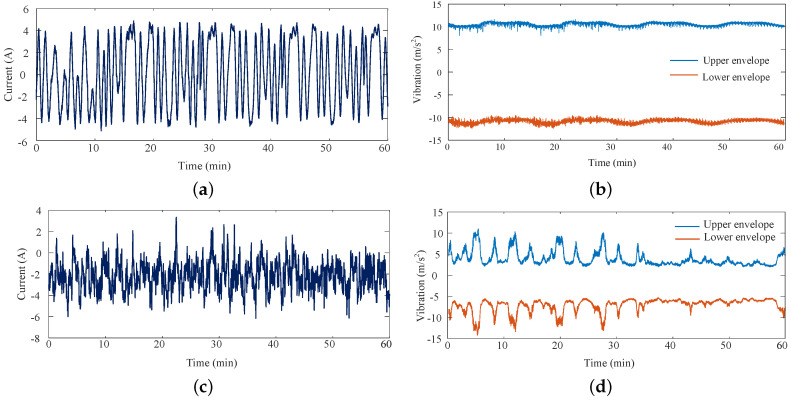
Transformer neutral DC and vibration. (**a**) Time-magnitude of neutral DC from 05:00 to 06:00. (**b**) Time-magnitude of vibration from 05:00 to 06:00.(**c**) Time-magnitude of neutral DC from 16:00 to 17:00. (**d**) Time -magnitude of vibration from 16:00 to 17:00.

**Figure 5:**
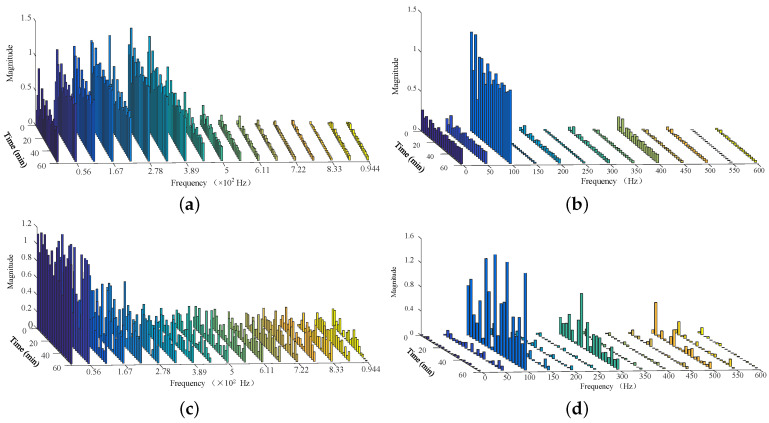
Transformer neutral DC and vibration. (**a**) Time-frequency of neutral DC from 05:00 to 06:00. (**b**) Time-frequency of vibration from 05:00 to 06:00. (**c**) Time-frequency of neutral DC from 16:00 to 17:00. (**d**) Time -frequency of vibration from 16:00 to 17:00.

By using the proposed analysis method, the time-magnitude distributions of neutral DC and vibration were calculated and are shown in Figure 4. Figure 4a,b are the time-magnitudes of the transformer-neutral DC and vibration values from 05:00 to 06:00. Figure 4c,d are the time-magnitudes from 16:00 to 17:00. Select 3 min as the framing time. The time-frequency distributions of neutral DC and vibration are converted and shown in Figure 5. Figure 5a,b are the time-frequency of transformer-neutral DC and vibration from 05:00 to 06:00. Figure 5c,d are the time-frequencies from 16:00 to 17:00.

When no train operates in the metro system, the neutral DC and vibration magnitudes are steady. When dynamic trains operate from 16:00 to 17:00, the magnitudes of neutral DC and vibration fluctuate obviously. Moreover, because the upper and lower envelopes of vibration are similar, the upper envelope is selected as the magnitude characteristic. The time-magnitude characteristics of the neutral DC and vibration are the one-dimensional data with time. Thus, the magnitude characteristics were selected as the final magnitude characteristics.

Obviously, when no train operates in the metro system, even-frequency components are the main frequencies, and the odd-frequency components are small enough to ignore. When dynamic trains operate from 16:00 to 17:00, the low-frequencies of neutral DC increase obviously. The odd-frequency components and frequency complexity are increased totally.

**Figure 6:**
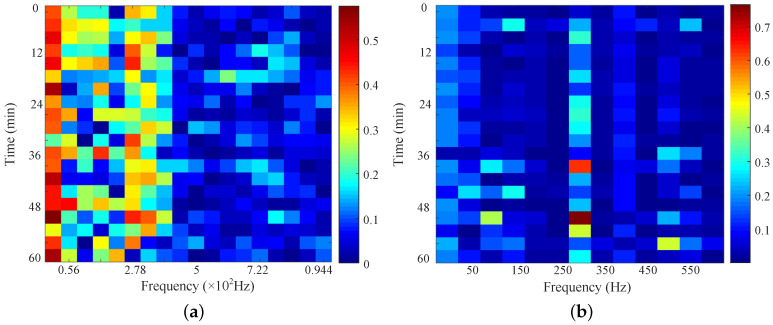
The frequency characteristic difference with and without stray current. (**a**) Frequency characteristic difference of neutral DC. (**b**) Frequency characteristic difference of vibration.

The time-frequency characteristics are complex. The frequency characteristics of neutral DC and vibration need to be screened and merged using the proposed analysis method. The time-frequency characteristics are normalized based on function (2), and the differences are calculated. The differences in the frequency characteristics are shown in Figure 6.

**Figure 7:**
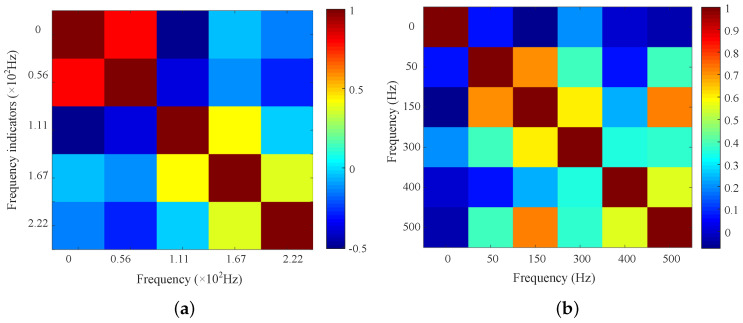
Pearson correlation coefficients of primary characteristics. (**a**) Correlation coefficients among neutral DC characteristics. (**b**) Correlation coefficients among vibration characteristics.

The average of the difference matrices serves as the threshold. Select those characteristics with greater-than-average differences as the primary characteristics. The primary frequency characteristics of neutral DC include 0 Hz, 0.0056 Hz, 0.0111 Hz, 0.0167 Hz, and 0.022 Hz. The primary frequency characteristics of vibration include 0 Hz, 50 Hz, 150 Hz, 300 Hz, 400 Hz, and 500 Hz. Then, the Pearson correlation coefficient among the primary characteristics is calculated. The Pearson correlation coefficients are calculated based on function (3) and are shown in Figure 7.

In Figure 7a, the correlation coefficient between 0 Hz and 0.0056 Hz is 0.81. The correlation coefficient between 0 Hz and 0.0111 Hz is −0.52. The correlation coefficients among the other frequency components are all less than 0.6. That is, the characteristics of 0 Hz and 0.0056 Hz are similar and can be combined to simplify the indicator number. In Figure 7b, the correlation coefficient between 50 Hz and 100 Hz is 0.67. The correlation coefficient between 100 Hz and 500 Hz is 0.72. The correlation coefficient between 300 Hz and 400 Hz is 0.60. The correlation coefficients among the other frequency components are all less than 0.6. The characteristics of 50 Hz, 100 Hz, and 500 Hz are similar, and the characteristics of 300 Hz and 4500 Hz are similar. Thus, to decrease the number of indicators, a hierarchical clustering method is used to combine similar characteristic indicators.

The hierarchical clustering method is used to combine the characteristics with a correlation of greater than 0.6. The final frequency characteristics of neutral DC include (0, 0.0056 Hz), 0.0111 Hz, 0.0167 Hz, and 0.022 Hz. The final frequency characteristics of neutral DC include 0, (50 Hz, 150 Hz, and 500 Hz), and (300 Hz and 400 Hz).

### 4.2. Assessment of DC Bias Risk

According to the extracted indicators of neutral DC and vibration, the indicator quantization results are calculated based on function (6). In the calculation process, the threshold values of the indicators are determined based on the average values of the indicators from 5:00 to 7:00 because, during this time, the number of trains increases from zero and gradually increases.

The weights of the extracted indicators are calculated based on functions (7)–(9). The entropy and weight of the indicators are shown in Table 2. Then, by using the indicator weights, the assessment scores can be calculated by using function (10). Then, the assessment results are obtained according to the DC bias risk level classifications shown in Table 1.

In the field experience method, the transformer DC bias risk is evaluated by using neutral DC magnitude. Commonly, the transformer DC bias risk exists when the neutral DC is larger than 5 A. By comparing the proposed assessment method and the field expertise method, the assessment results of transformer DC bias risk based on the test data from 16:00 to 17:00 are shown in Table 3. Select 3 min for the assessment time. There are 20 assessment results based on two methods.

When comparing the evaluation results of the two methods, the results of the proposed method are more detailed. The evaluation results of the field experience method are mostly of no risk. However, from 16:00 to 17:00, the stray currents flow into the transformers, and the DC bias risk exists. Thus, the results of the field experience method are not accurate. While using the proposed method, the evaluation results are low-level risk and more realistic. Therefore, the proposed method is more suitable for evaluating the transformer DC bias caused by metro stray currents.

## 5. Conclusions

Metro stray currents flowing into a transformer can cause transformer DC bias risk. In order to evaluate the DC bias risk caused by stray currents, characteristic extraction and assessment methods are proposed in this work. By using the proposed method, the primary characteristics are analyzed by comparing the magnitude and frequency distributions of neutral DC and vibration with and without the stray current effect. Then, the characteristic indicators are extracted by using Pearson’s correlation analysis and hierarchical clustering methods. EWM is used to weight the indicators and assess the level of DC bias risk. Based on the neutral DC and vibration data of a transformer, the characteristic extraction method proposed in this paper is applied. The magnitude and frequency indicators are obtained, and the entropy and weight of each indicator are calculated by EWM. Moreover, the evaluation results of the field experience method and the proposed method are compared. The comparison results show that the assessment results of the proposed method are realistic and accurate. In conclusion, the proposed method is suitable for the evaluation of the DC bias of any type of transformer affected by metro stray currents.

## Figures and Tables

**Table 1:** Indicators extracted from the test data.

DC Bias Risk Level	No Risk	Low Level	Middle Level	High Level
Assessment scores	(0, 0.01]	(0.01, 0.1]	(0.1, 0.5]	(0.5, 1)

**Table 2:** Indicators extracted from the test data.

Number	Type	Indicators	Entropy	Weight
1	Neutral DC	Magnitude indicator $M_{1}$	0.946	0.082
2		Frequency indicator $F_{0+0.0056}$	0.945	0.088
3		Frequency indicator $F_{0.0111}$	0.856	0.083
4		Frequency indicator $F_{0.0167}$	0.894	0.103
5		Frequency indicator $F_{0.022}$	0.867	0.127
6	Vibration	Magnitude indicator $M_{2}$	0.888	0.068
7		Frequency indicator $F_{0}$	0.780	0.149
8		Frequency indicator $F_{50+150+500}$	0.806	0.151
9		Frequency indicator $F_{300+400}$	0.834	0.147

**Table 3:** Evaluation results of transformer DC bias risk.

Framing Time No.	Field Experience	Proposed Method	Framing Time No.	Field Experience	Proposed Method
1	No risk	Low-level risk	11	Existed risk	Low-level risk
2	Existed risk	Middle-level risk	12	Existed risk	Low-level risk
3	No risk	Low-level risk	13	Existed risk	Low-level risk
4	No risk	Low-level risk	14	No risk	Low-level risk
5	No risk	Low-level risk	15	No risk	No risk
6	No risk	Low-level risk	16	Existed risk	Low-level risk
7	No risk	Low-level risk	17	No risk	Low-level risk
8	Existed risk	Middle-level risk	18	Existed risk	Low-level risk
9	No risk	No risk	19	No risk	No risk
10	No risk	Low-level risk	20	No risk	Low-level risk

## Data Availability

The original contributions presented in the study are included in the article, further inquiries can be directed to the corresponding author.
